# A novel conjugated polymer synthesized *via* a noble metal-free catalyst in photothermal therapy of hepatocellular carcinoma mediated by second near-infrared (NIR-II) laser

**DOI:** 10.1016/j.mtbio.2025.101488

**Published:** 2025-01-13

**Authors:** Shengsheng Cui, Xinni Pan, Shanshan Fan, Cheng Cao, Yingao Jiao, Yanfei Fu, Jiaqi Niu, Shujin Lin, Jingmao Lao, Yanlei Liu

**Affiliations:** a Institute of Intelligent Health Diagnosis and Treatment, School of Sensing Science and Engineering, School of Electronic Information and Electrical Engineering, Shanghai Jiao Tong University, 800 Dongchuan Road, Shanghai, 200240, PR China; b Gastrointestinal Surgery, The First People's Hospital of Qinzhou, Qinzhou, 535000, PR China; c National Engineering Center for Nanotechnology, Shanghai, 200240, PR China

**Keywords:** Conjugated polymer, Phenazine ring fusion reaction, Photothermal therapy

## Abstract

Photothermal therapy (PTT) utilizes photothermal materials to convert light energy into heat under external light irradiation, effectively killing cancer cells. Therefore, the efficacy of PTT is largely determined by the photothermal conversion efficiency of the material. In this study, we developed a novel ladder-type conjugated polymer, PPAPA, *via* a phenazine ring fusion reaction. PPAPA exhibits a high photothermal conversion efficiency of 75.2 % under 1064 nm laser irradiation, comparable to the benchmark organic photothermal agent SWCNT. Notably, the synthesis of PPAPA avoids the use of noble metal catalysts, eliminating potential biotoxicity caused by residual catalysts and ensuring optimal photothermal stability and efficiency. Furthermore, PPAPA demonstrates efficient photothermal conversion under near-infrared II (NIR-II) 1064 nm laser irradiation, enabling deeper tissue penetration and reduced tissue absorption. This work comprehensively investigates the photothermal properties of PPAPA and evaluates its efficacy in tumor PTT, demonstrating its potential as a novel and effective therapeutic strategy for cancer treatment, offering new hope for patients.

## Graphical abstract

**Figure:**
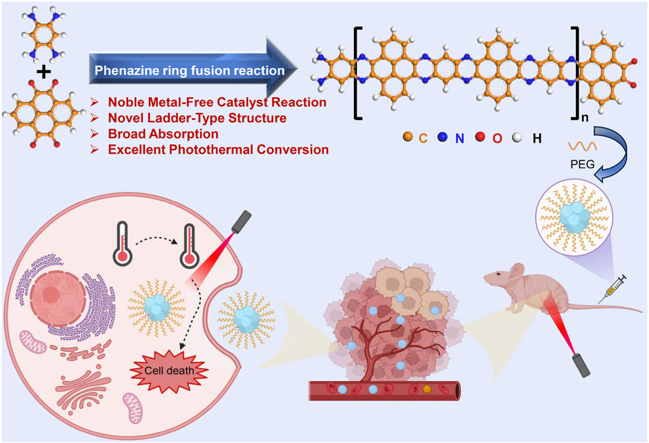

## 1 Introduction

Photothermal therapy (PTT) offers a promising alternative or supplement to traditional cancer therapy by utilizing light to elevate tissue temperature [1]. Its inherent advantages include spatially targeted light irradiation, minimal invasiveness, simple procedures, short treatment duration, and quick recovery, attracting extensive research interest [[2], [3], [4], [5]]. Numerous inorganic photothermal agents (PTAs) have been developed, including gold nanomaterials [[6], [7], [8], [9]], layered transition metal dichalcogenides [[10], [11], [12], [13]], carbon nanomaterials (carbon nanotubes, graphene and their derivatives) [[14], [15], [16], [17]], quantum dots [[18], [19], [20], [21]], upconversion nanocomposites [[22], [23], [24]], black phosphorus quantum dots [[25], [26], [27], [28]], and others [29,30]. These PTAs demonstrate favorable absorbance features, excellent photothermal conversion efficiencies, and good photostability. However, their slow excretion and retention in major organs of the reticuloendothelial system, particularly the liver and spleen, raise concerns about long-term toxicity, limiting their further clinical applications [31].

In recent years, these emerging organic PTAs have attracted significant attention [[32], [33], [34], [35], [36]]. Conjugated polymers, composed of completely benign and bio-inert components, offer an alternative to inorganic PTAs, circumventing toxicity concerns while exhibiting comparable or even superior properties [[37], [38], [39]]. Polyaniline [40], polypyrrole [41], poly(3,4-ethylenedioxythiophene) [42], polydopamine [43], and donor-acceptor (D-A)-structured conjugated polymers [44] have been reported for PTT. Their synthesis typically includes chemical oxidative polymerization [40,45], electropolymerization [46], Suzuki coupling [[47], [48], [49]], Heck coupling [50], Sonogashira coupling [51], and Stille coupling [[52], [53], [54]]. However, most coupling reactions require noble metal catalysts, which may remain in the synthesized organic PTAs, and cause side effects in biomedical applications, while also influencing their photothermal properties. Among these methods, Stille coupling, which involves coupling different electron donors and acceptors to synthesize D-A-structured conjugated polymers, has dominated in recent years, with limited examples of other reaction types [[55], [56], [57], [58], [59]]. Recently, Liang et al. reported the modified click condensation reaction for facile synthesis of conjugated polymers with photothermal heating-up speeds comparable to those of star material single-wall carbon nanotubes (SWCNTs) [60]. More recently, Tang et al. adopted cycloaddition-retroelectrocyclization click reaction to synthesize high-performance organic PTAs [61]. Therefore, it is highly desirable to develop simpler and more efficient reaction approaches for constructing conjugated polymers.

**Scheme 1:**
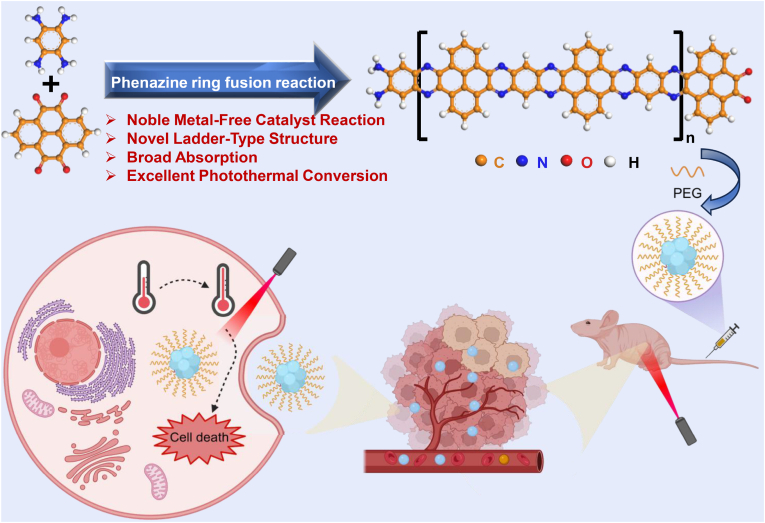
Schematic illustration of the synthesis process and photothermal effect of poly-phenanthrol-phenazine nanoparticles (PPAPA NPs).

Phenazine ring fusion reaction is a powerful tool in organic synthesis. It involves the condensation of quinone and amine acid derivatives at high temperatures with acid catalysts [62]. To date, this reaction has been utilized to synthesize different conjugated microporous polymers and covalent organic frameworks for applications such as photo/electrocatalysis [63], supercapacitors [64], batteries [65], and water purification [66]. However, its application in synthesizing organic PTAs remains limited. Furthermore, existing organic PTAs synthesized through abovementioned various coupling methods often suffer from oxidative decomposition due to their C-C single bond-based extended conjugated π-systems, leading to photobleaching and subsequent insufficient photothermal conversion efficiency (PTCE) [67]. To address such challenge, we present a novel ladder-type poly-phenanthrol-phenazine (PPAPA) synthesized *via* phenazine ring fusion reaction using 1,2,4,5-Tetraaminobenzene tetrahydrochloride (TAB) and 4,5,9,10-Pyrenetetrone (PT). Characterization of this ladder-type polymer was performed using nuclear magnetic resonance (NMR), mass spectrometry (MS), and steady-state spectroscopies. The resulting PPAPA exhibits excellent absorption in the ultraviolet–visible–near infrared (UV–Vis–NIR) region, indicating its potential as organic PTAs. Compared to SWCNTs, PPAPA demonstrates a superior photothermal heating-up speed. Furthermore, its photothermal conversion efficiency (η) under 1064 nm laser irradiation is calculated as 75.2 %, based on the cooling curve, falling within the photothermal conversion in NIR-II optical window, indicating deeper tissue penetration, lower scattering, and reduced tissue absorption. Through a routine nanoprecipitation method, we successfully coated the hydrophobic PPAPA with 1,2-distearoyl-sn-glycero-3-phosphoethanolamine-N-[methoxy(polyethylene glycol)] (DSPE-mPEG) to obtain highly stable, physiologically dispersed, and photostable nanoparticles (PPAPA NPs). Our as-prepared PPAPA NPs show negligible dark toxicity but excellent tumor cell killing effect under 1064 laser irradiation. The effective tumor-suppressive effect of PPAPA nanoparticles through NIR-II photothermal treatment was also further verified by *in vivo* experiments (Scheme 1). Our results suggest that PPAPA NPs could potentially open new avenues for tumorous photothermal therapy.

## 2 Results

### 2.1 Facile synthesis and physical characterizations of PPAPA

**Fig. 1:**
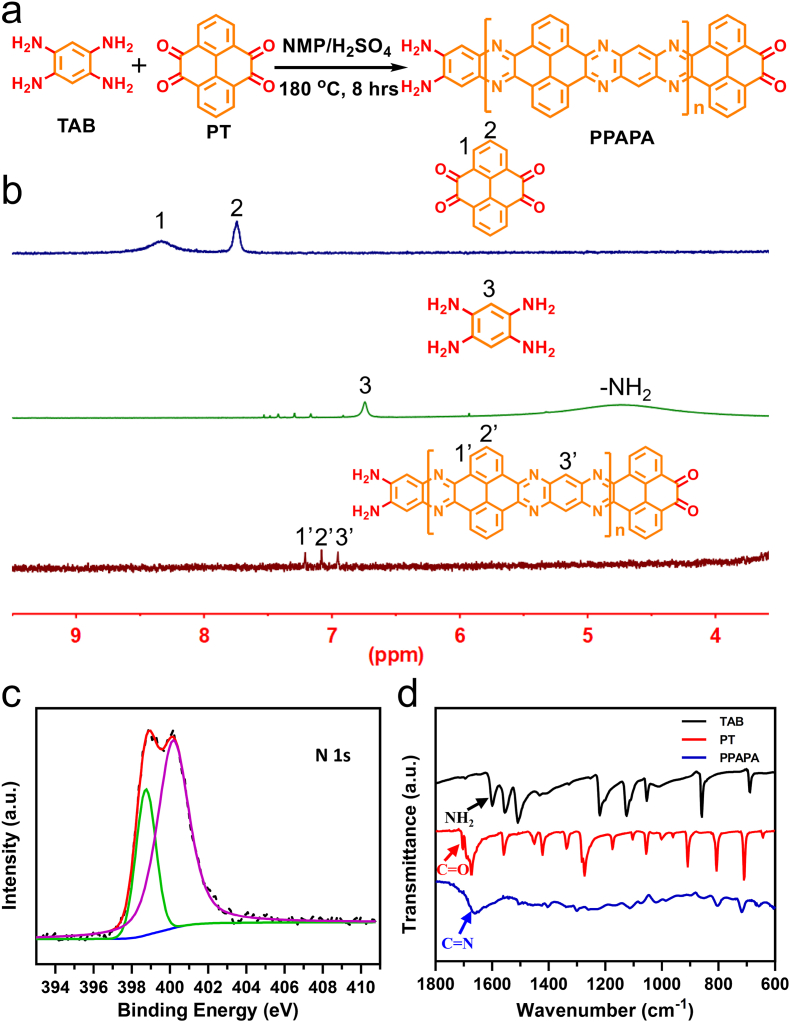
Synthesis and Characterization of Poly-phenanthrol-phenazine (PPAPA) (a) Schematic illustration of the synthesis procedures for PPAPA; (b) ^1^H NMR spectra of 4,5,9,10-pyrenetetrone (PT, top), 1,2,4,5-tetraaminobenzene tetrahydrochloride (TAB, middle), and PPAPA (bottom) in DMSO-*d*_6_; (c) High-resolution N 1s XPS spectrum of PPAPA; (d) FTIR vibration spectra for TAB, PT, and PPAPA.

The conjugate ladder-type polymer PPAPA was synthesized using the modified phenazine ring fusion reaction (Fig. 1a). Detailed synthesis steps were described in the Supporting Information. Briefly, equivalent amounts of 1,2,4,5-Tetraaminobenzene tetrahydrochloride (TAB) and 4,5,9,10-Pyrenetetrone (PT) were mixed at 8.8 mM in NMP with H_2_SO_4_ at 180 °C for 8 h. The reaction was quenched with water, yielding black precipitates of PPAPA in large quantity (yield: 57.7 %). This high yield suggests a rapid reaction rate for this phenazine ring fusion reaction. The crude product was filtrated and rigorously washed using a Soxhlet extractor with water and methanol for 24 h to remove the catalyst and remaining starting materials. After drying under vacuum, pure black powder product was obtained. The successful synthesis of PPAPA was confirmed. In the ^1^H NMR spectrum, the broad peak at 4.73 ppm corresponding to the hydrogen of the TAB's amidogen disappeared in PPAPA due to the phenazine ring fusions reaction between aryl diamines and aryl diketones (Fig. 1b and Figs. S1–S3). Additionally, the aromatic protons of PPAPA (site 1′ and 2′) shifted towards the high field compared to the aromatic protons of PT (site 1 and 2) due to the electron-donating effect of the amidogen. The successful formation of the quinoxaline structure was confirmed by X-ray photoelectron spectroscopy (XPS) spectra in Fig. 1c and S4, where a newly appeared N 1 s peak at 398.7 eV of PPAPA was assigned to the *sp*^*2*^-hybridized nitrogen in the newly formed C-N-C structures on the quinoxaline structure (Fig. 1c). Furthermore, matrix-assisted laser desorption ionization-time of flight mass spectrometry (MALDI-TOF MS) analysis of the PPAPA revealed a number-average molecular weight of approximately 19,000 with a polydispersity (PDI) of 1.077 (Fig. S5). This result is in good agreement with the gel permeation chromatography (GPC) results in Fig. S6 (a number-average molecular weight of approximately 23,105 with a PDI of 1.038). The functional groups in the initial materials and target product were also characterized by fourier transform infrared spectroscopy (FT-IR) (Fig. 1d), where the disappearance of TAB's amino bending vibration peak at 1599 cm^−1^ and PT's C=O stretching vibration peak at 1704 cm^−1^, along with the appearance of PPAPA's aromatic rings C=N stretching vibration peak at 1660 cm^−1^, indicated the successful occurrence of the phenazine ring fusion reaction. Thus, the ^1^H NMR spectra, XPS spectra, MALDI-TOF MS, and FT-IR all confirmed the successful synthesis of PPAPA.

**Fig. 2:**
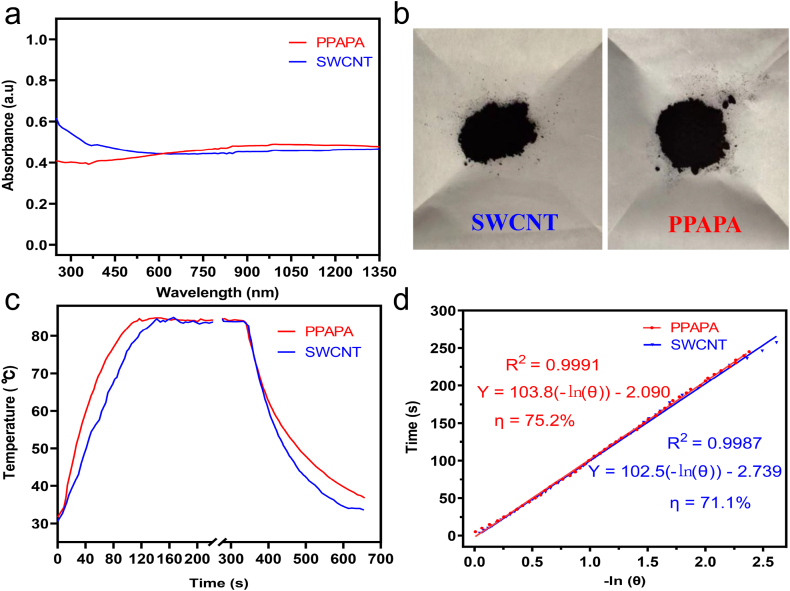
(a) UV–Vis–NIR absorption spectra of 0.2 mg/mL PPAPA and SWCNT suspensions; (b) Photographs of PPAPA and SWCNT; (c) Temperature curve under 1064 nm laser irradiation (light source removed at approximately 220 s); (d) Linear correlation between cooling times and the negative natural logarithm of driving force temperatures for PPAPA and SWCNT.

UV–Vis–NIR spectra of PPAPA and SWCNT (Fig. 2a) revealed a broad absorption band from 250 to 1350 nm for both materials, indicating their ability to efficiently absorb and utilize light energy across a wide range. This is consistent with the observation that both PPAPA and SWCNT appear as black powders (Fig. 2b). Ultraviolet photoelectron spectroscopy (UPS) analysis (Fig. S7) revealed an ionization potential of −6.8 eV for PPAPA, suggesting a good oxidative stability. These characterizations indicated the promising photothermal properties of our conjugate ladder-type polymer PPAPA.

To demonstrate these photothermal properties, SWCNT, a benchmark photothermal biomedical material known for its excellent thermal conductivity, was adopted for parallel study. PPAPA and SWCNT suspensions (both 0.2 mg/mL) exhibited a rapid temperature increase when exposed to a 1064 nm laser with a power density of 2.0 W/cm^2^, reaching their temperature plateaus at 160 s (Fig. 2c). Notably, PPAPA displayed a comparable or even faster heating-up speed compared to SWCNT. Under these conditions, the maximum photothermal temperature reached by PPAPA was 84.3 °C, which is equivalent to the 84.2 °C reached by SWCNT. Based on the cooling curve of the PPAPA solution, we then calculated the photothermal conversion efficiency (η) of PPAPA as 75.2 % according to the previously reported method (Fig. 2d). We propose that the better NIR performance of PPAPA is due to its higher absorption coefficient at NIR region than SWCNT. To the best of our knowledge, this photothermal conversion efficiency is higher than that of most previously reported conjugated polymers PTAs [44,49,52,53,60,68]. Therefore, we envision that PPAPA would be a promising candidate for biomedical applications considering its SWCNT-comparably high photothermal capability.

### 2.2 Photothermal properties of PPAPA NPs

**Fig. 3:**
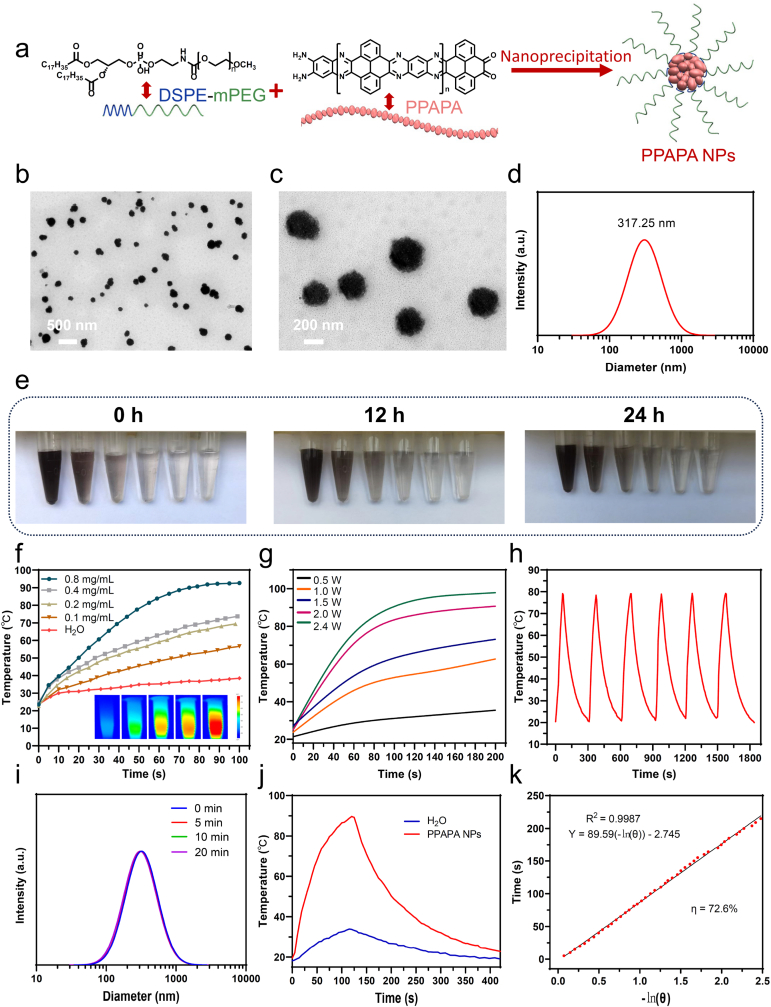
(a) Schematic illustration of the preparation process for PPAPA NPs; (b) Transmission electron microscopy (TEM) image of PPAPA NPs; (c) Magnified TEM image of PPAPA NPs; (d) Dynamic light scattering (DLS) measurement of PPAPA NPs; (e) Optical images of PPAPA NPs dispersed in water at different concentrations during various times (From left to right, the concentration of the solutions is 0.8, 0.4, 0.2, 0.1, 0.05, and 0.025 mg/mL in order); (f) Temperature-changes curves of PPAPA NPs under 1064 nm laser excitation at different concentrations with the same power density of 1.5 W/cm^2^; inset: the corresponding thermographs; (g) Temperature change curves of PPAPA NPs upon exposure to the 1064 nm NIR laser at a power density of 0.5, 1.0, 1.5, 2, or 2.4 W/cm^2^; (h) Temperature elevation curves of PPAPA NPs over six cycles of 1064 nm NIR laser on/off irradiation at a power density of 2.4 W/cm^2^; (i) Dynamic light scattering (DLS) measurement of PPAPA NPs after laser irradiation (1064 nm, 1 W/cm^2^) for different times; (j) The photothermal heating and cooling curves of PPAPA NPs (0.8 mg/mL) and water under the 1064 nm laser irradiation; (k) Linear correlation between cooling times and the negative natural logarithm of driving force temperatures for PPAPA NPs.

To investigate the potential of PPAPA for cancer treatment *via* PTT, we encapsulated PPAPA into water-soluble polymer nanoparticles (PPAPA NPs) using DSPE-mPEG, an amphiphilic diblock copolymer commonly used in drug delivery systems (Fig. 3a). Transmission electron microscopy (TEM) revealed a spherical structure morphology with a diameter of about 260 nm for the nanoparticles (Fig. 3b and c) Dynamic light scattering (DLS) showed an average hydrodynamic diameter of 317.25 nm for PPAPA NPs (Fig. 3d). The nanoprecipitation method yielded a dark brown monodispersion of PPAPA NPs in water, which remained stable for at least 24 h (Fig. 3e). Moreover, PEGylated PPAPA nanoparticles could be well dispersed in physiological buffers without showing any obvious aggregation (Fig. S8). Furthermore, the average hydrodynamic diameter of PPAPA NPs in physiological buffers matched the results in aqueous solutions (Fig. S9).

To assess the photothermal properties of PPAPA NPs, its UV–Vis–NIR spectra was first tested, revealing that PPAPA NPs also have a wide absorption band in the range of 250–1350 nm like PPAPA (Fig. S10), proving that 1064 nm laser can be used for photothermal therapy. Then, the 1064 nm laser at a power density of 1.5 W/cm^2^ was used to irradiate PPAPA NPs solutions of different concentrations. The temperature changes in the solution were positively correlated with the concentration of the PPAPA NPs. After 100 s irradiation, PPAPA NPs solutions with concentrations of 0.8, 0.4, 0.2, and 0.1 mg/mL reached temperatures of 92.6 °C, 73.7 °C, 69.4 °C, and 56.6 °C, respectively (Fig. 3f). In contrast, deionized water only reached a temperature of 38.5 °C under the same conditions. PPAPA NPs solutions with a fixed concentration of 0.2 mg/mL were also irradiated with different power density: 0.5, 1, 1.5, and 2 W/cm^2^. The temperature of the solution increased rapidly with increasing laser power, and then slowly reached a plateau. As the laser power increased, both the heating rate and the maximum temperature reached also increased. A laser power of at least 1.0 W/cm^2^ was required for the solution temperature to reach above 42 °C (Fig. 3g). This power density was chosen for subsequent *in vitro* experiments to achieve an effective photothermal therapeutic effect while ensuring safety. Subsequently, the photothermal stability of PPAPA NPs was further demonstrated by six rounds of NIR laser on/off irradiation (1064 nm laser, 2.4 W/cm^2^, laser on for 70 s, then off until the solution naturally cooled down to room temperature). After six cycles, the temperature elevation of the PPAPA NPs solution remained consistent with the first cycle, indicating excellent photothermal stability (Fig. 3h). In addition, we continuously monitored the average hydrodynamic diameter of PPAPA NPs after laser irradiation (1064 nm, 1 W/cm^2^) for different durations (5, 10, and 20 min) and found no significant changes, further verifying the photostability of the PPAPA NPs (Fig. 3i). Compared to water, PPAPA NPs rapidly heated up to 90 °C after 125 s of laser irradiation (Fig. 3j), which can cause irreversible tumor cell necrosis. Its relatively slow cooling process contributes to sustained photothermal therapy efficacy, further enhancing its effectiveness. Based on the cooling curve in Fig. 3j, we then calculated the photothermal conversion efficiency (η) of PPAPA NPs as 72.6 % (Fig. 3k), which is slightly lower than PPAPA (η = 75.02 %). This may be due to the weakened absorption caused by PEG encapsulation.

### 2.3 Cellular uptake of PPAPA NPs

**Fig. 4:**
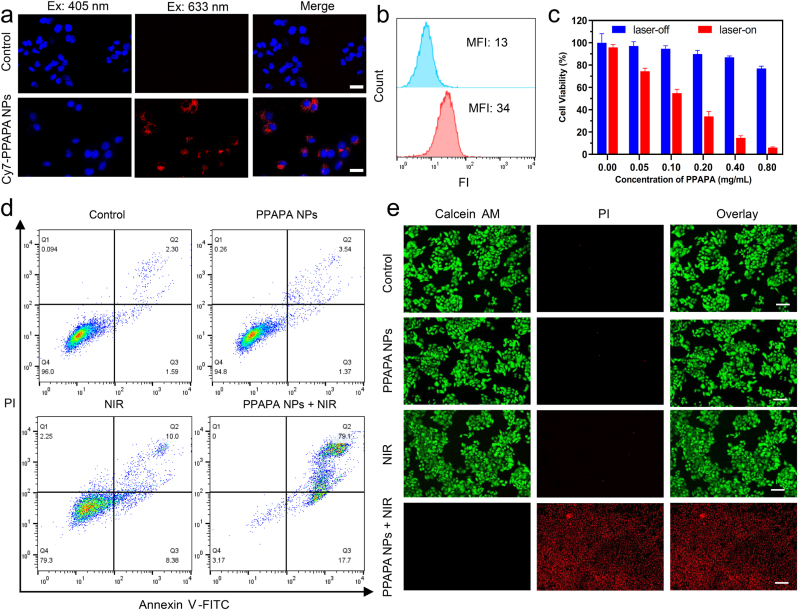
Assessment of cellular uptake efficiency of PPAPA NPs and therapeutic efficacy of PPAPA NPs *in vitro*. (a) Representitive CLSM images of BEL-7402 cells incubated with PBS or Cy7-PPAPA NPs (0.2 mg/mL) for 12 h. Cells incubated with Cy7-PPAPA NPs showed significant intracellular red fluorescence signals. Nuclei were stained blue with DAPI. Scale bars: 25 μm. (b) Flow cytometric analysis of Bel-7402 cells incubated with PBS or Cy7-PPAPA NPs (0.2 mg/mL) for 12 h. (c) Cell viabilities of BEL-7402 cells incubated with the PPAPA NPs at different concentrations with (red columns) or without (blue columns) the 1064 nm laser illumination (1.0 W/cm^2^) for 5 min. (d) Flow cytometric analysis of the apoptosis of Bel-7402 cells after receiving different treatments (Control, PPAPA NPs, NIR, PPAPA NPs + NIR). (e) Live/dead imaging of Bel-7402 cells treated with the corresponding four groups in (d). Green: live; red: dead. Scale bar: 100 μm. (For interpretation of the references to colour in this figure legend, the reader is referred to the Web version of this article.)

Effective cellular uptake of nanoparticles is essential for efficient therapy and is influenced by factors such as the size and shape of the material, as well as the surface chemistry. In this study, the uptake of PPAPA NPs by BEL-7402 cells was validated using two methods. Confocal laser scanning microscopy (CLSM) was used to assess the efficiency of cellular uptake of PPAPA NPs. Before that, Cy7, a near-infrared (NIR) sensitive dye, was co-encapsulated with PPAPA using the same nanoprecipitation method to form Cy7-labeled PPAPA nanoparticles (Cy7-PPAPA NPs) to provide an indication of the location and amount of PPAPA NPs in the cells. After co-incubating BEL-7402 cells and Cy7-PPAPA NPs for 12 h, the cell nuclei were stained blue with DAPI. Compared to the control group, numerous red fluorescent dots were observed in the cytoplasm of PPAPA NPs-treated cells (Fig. 4a). The dense bright red foci detected in the cytoplasm responded to the large aggregation of PPAPA NPs in the perinuclear and cytoplasmic regions, confirming the excellent internalization and uptake of PPAPA NPs by BEL-7402 cells. To quantify the uptake capacity of BEL-7402 cells, flow cytometry was employed to detect the intracellular fluorescence signals. After 12 h of incubation, the fluorescence signals of the cells in the PPAPA NPs-treated group were significantly increased compared to the control group, with their mean fluorescence intensity being 2.6 times higher than that of the untreated control group (Fig. 4b).

### 2.4 *In vitro* photothermal therapy efficacy and cytotoxicity of PPAPA NPs

Next, *in vitro* photothermal therapy (PTT) efficacy and cytotoxicity of PPAPA NPs were evaluated using CCK-8 assays and cell viability assessments. Varying concentrations of PPAPA NPs solutions up to 0.4 mg/mL did not affect the growth of BEL-7402 cells, with relative cell viability remaining above 86 % (Fig. 4c). The similar conclusion was confirmed in L02 cells (Fig. S11). However, upon exposure to 1064 nm laser irradiation for 2 min, cellular activity significantly decreased to approximately 15 % in the 0.4 mg/mL PPAPA NPs condition. Flow cytometry analysis of cell apoptosis analysis further confirmed these findings. The percentage of apoptotic cells increased from 3.54 % to 79.1 % upon exposure to 1064 nm NIR laser in BEL-7402 cells co-incubated with PPAPA NPs (Fig. 4d). Additionally, to visually evaluate the NIR-II PTT impact of PPAPA NPs *in vitro*, cells receiving various treatments were stained with calcein-AM/PI double dye and imaged using confocal laser scanning microscopy. In the control, PPAPA NPs-only and NIR-only groups, BEL-7402 cells exhibited bright green fluorescence, indicating minimal cell death (Fig. 4e). This indicates that neither PPAPA NPs treatment alone nor 1064 nm laser irradiation alone could effectively kill tumor cells. These results also highlight the better biocompatibility of PPAPA NPs, which is advantageous for further anti-tumor treatment. In contrast, cells treated with PPAPA NPs and 1064 nm laser displayed substantial patches of red fluorescence (Fig. 4e), indicating significant cell death. These outcomes demonstrate the potential of PPAPA NPs as effective NIR-II PTT agents for cancer treatment.

### 2.5 *In vivo* fluorescent imaging

**Fig. 5:**
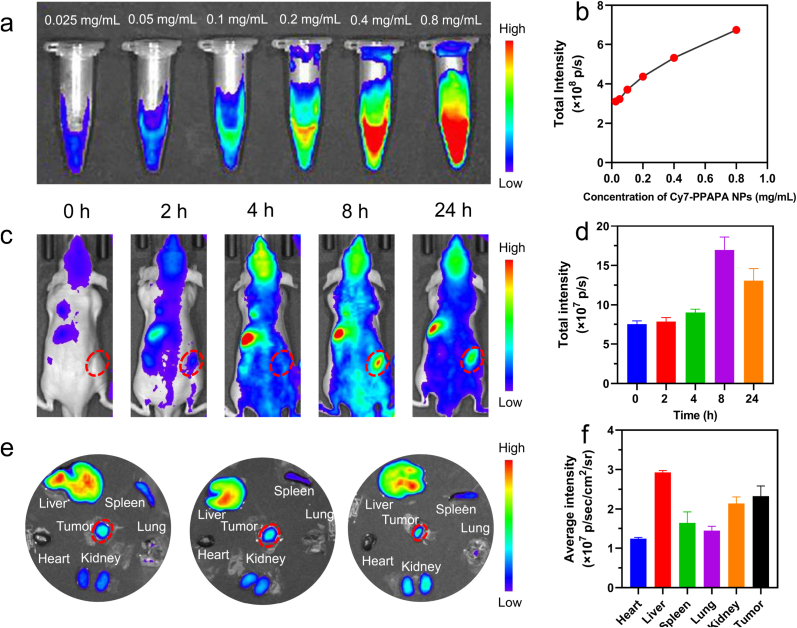
*In vivo* distribution of PPAPA NPs. (a) *In vitro* fluorescence imaging of Cy7-PPAPA NPs with different concentration. (b) The corresponding fluorescence intensity analysis of Cy7-PPAPA NPs with different concentration. (c) *In vivo* images of BEL-7402 tumor-bearing nude mice at different time intervals after injection of Cy7-PPAPA NPs. (d) The fluorescence intensity of the tumor area at different time intervals after injection of Cy7-PPAPA NPs. (e) *Ex vivo* images of major organs excised from the mice injected with Cy7-PPAPA NPs. (f) Average fluorescence intensity of tumors and major organs. Data are expressed as the mean ± SD (n = 3).

Achieving precise control over the temporal distribution of nanoparticles at tumor sites is essential for determining the optimal therapeutic window. To investigate the *in vivo* distribution of PPAPA NPs, we conducted fluorescence imaging studies in nude mice with xenografted tumors. The Cy7-PPAPA NPs were used to facilitate tracking within the mice. Initially, imaging was conducted on materials of varying concentrations, revealing a positive correlation between fluorescence intensity and material concentration (Fig. 5a and b). Subsequently, following intravenous injection of Cy7-PPAPA NPs into the mice *via* the tail vein, NIR fluorescence imaging was executed at predetermined time intervals. The results showed that at the tumor site, the initial detection of the fluorescence signal occurred at the 2 h mark. Thereafter, the intensity of the signal escalated progressively, culminating in a maximum at the 8 h interval post-injection (Fig. 5c and d). Consequently, this time point was selected as the commencement of treatment. Post-mortem imaging of the extracted organs showed that PPAPA NPs were still present in the spleen, liver, and kidneys after 24 h. Particularly, the liver and tumor regions displayed the most significant accumulation of PPAPA NPs relative to other tissues examined (Fig. 5e and f).

### 2.6 *In vivo* therapeutic performance of PPAPA NPs

**Fig. 6:**
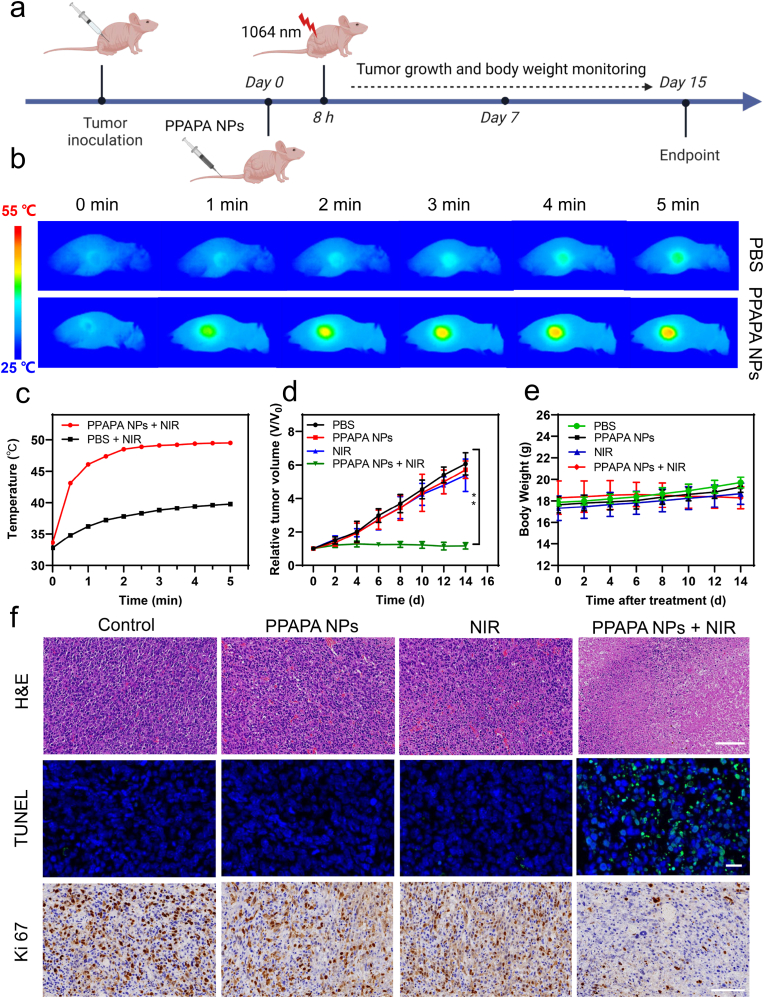
Therapeutic efficacy of PPAPA NPs *in vivo*. (a) Schematic illustration of the tumor therapeutic profile. (b) Thermographs and (c) corresponding temperature changes on the tumor sites of mice bearing Bel-7402 xenograft tumors under 1064 nm laser irradiation at 1.0 W/cm^2^ for 5 min after intravenous injection of PPAPA NPs (10 mg/mL, 100 μL) for 8 h. (d) Tumor growth inhibition curves and (e) body weight changes for various treatments (n = 5). Results are presented as mean ± S.D., p > 0.05; ∗p < 0.05; ∗∗p < 0.01, analyzed by Student's t-test. (f) Representative hematoxylin-eosin, TUNEL staining and Ki 67 staining of tumor sections of each treatment group, Scale bar: (100 μm for H&E staining; 20 μm for TUNEL staining; 100 μm for Ki 67 staining).

Encouraged by the *in vitro* results, we validated the *in vivo* photothermal therapeutic feasibility of PPAPA NPs using BEL-7402 xenograft tumor-bearing mice (Fig. 6a). Mice were randomized into four groups (n = 5) with similar tumor volumes (approximately 100 mm^3^): (1) control, (2) PPAPA NPs only, (3) NIR only, and (4) PPAPA NPs + NIR. Mice in groups 1 and 3, mice received PBS injections, while mice in groups 2 and 4 received PPAPA NPs through tail vein injection. 8 h post-injection, tumor sites in mice from groups 3 and 4 were irradiated with a 1064 nm laser for 5 min (Fig. 6b). Following laser irradiation, the tumor temperature in the control group increased by approximately 7 °C–39.8 °C, while the PPAPA NPs + NIR group exhibited a rapid temperature increase to 49.5 °C (Fig. 6c). Such result demonstrates the outstanding *in vivo* photothermal conversion ability of PPAPA NPs and their potentials for photothermal ablation in anticancer therapy.

Tumor volume and body weight were monitored every two days to assess the *in vivo* photothermal therapy effectiveness of PPAPA NPs. The PPAPA NPs + NIR group showed significantly inhibited tumor growth, compared to the other groups, which exhibited exponential growthtrend (Fig. 6d–S12 and S13). Notably, no significant changes in body weight were observed across all groups during the experiment (Fig. 6e), indicating the safety and tolerability of our treatment. On day 15, all mice were euthanized and their subcutaneous tumor tissues and major organs (heart, liver, spleen, lungs, and kidneys) were dissected for H&E, TUNEL staining and Ki 67 staining. H&E staining of PPAPA NPs + NIR group exhibited marked nuclear consolidation, indicating a large area of necrosis in the tumor tissue. Conversely, no evident pathological damage was observed in the tumor tissues of the other groups. TUNEL staining confirmed substantial apoptosis in the PPAPA NPs + NIR group, whereas no noticeable apoptosis was observed in other treatment groups. which corresponded with the H&E staining. Ki-67 staining also showed the most significant proliferation inhibition in the PPAPA NPs + NIR group (Fig. 6f). These findings signify that NIR-II photothermal therapy with PPAPA NPs effectively destroys tumor cells and hinder tumor growth. H&E staining of major organs showed normal tissue structure and cellular morphology in all treatment groups compared to the control, with no obvious organ damage or toxic side effects such as inflammatory foci, indicated the excellent biological safety of PPAPA NPs (Fig. S14).

## 3 Conclusion

In this study, we report the synthesis of a novel conjugate ladder-type polymer, PPAPA, using a simple phenazine ring fusion reaction with sulfuric acid as the catalyst. This method eliminates the use of noble metal catalysts and avoids their potential contamination of the final product. PPAPA exhibits high photothermal conversion efficiency and excellent photothermal stability under NIR-II laser irradiation (1064 nm) due to its ladder-type conjugated structure. Surface modification of PPAPA with DSPE-mPEG yielded well-dispersed, size-uniform, and highly stable PPAPA NPs. These NPs demonstrated high biocompatibility in both *in vitro* and *in vivo* studies. Moreover, *in vitro* experiments revealed their effective killing ability against liver cancer cells. In a tumor-bearing mouse model, PPAPA NPs effectively accumulated at the tumor site and generated significant heat under 1064 nm laser irradiation, leading to efficient tumor cell killing and suppression of tumor growth. Therefore, PPAPA NPs hold great promise as a novel and effective photothermal therapy agent for cancer treatment.

## CRediT authorship contribution statement

**Shengsheng Cui:** Writing – original draft, Investigation, Funding acquisition, Data curation. **Xinni Pan:** Writing – original draft, Formal analysis, Data curation. **Shanshan Fan:** Methodology, Investigation. **Cheng Cao:** Investigation. **Yingao Jiao:** Methodology. **Yanfei Fu:** Visualization. **Jiaqi Niu:** Software, Methodology. **Shujin Lin:** Visualization, Funding acquisition. **Jingmao Lao:** Writing – review & editing, Supervision. **Yanlei Liu:** Writing – review & editing, Supervision, Funding acquisition.

## Declaration of competing interest

The authors declare that they have no known competing financial interests or personal relationships that could have appeared to influence the work reported in this paper.

## Data Availability

Data will be made available on request.
