# Supplementary material for: A novel conjugated polymer synthesized via a noble metal-free catalyst in photothermal therapy of hepatocellular carcinoma mediated by second near-infrared (NIR-II) laser
Source: Mater Today Bio. 2025 Jan 13;31:101488. doi: 10.1016/j.mtbio.2025.101488 (PMC11791355; doi:10.1016/j.mtbio.2025.101488)
Supplement: Multimedia component 1 [file mmc1.pdf]

Supporting Information

A Novel Conjugated Polymer Synthesized *via* a Noble Metal-Free Catalyst in Photothermal Therapy of Hepatocellular Carcinoma Mediated by Second Near-Infrared (NIR-II) Laser

Shengsheng Cui[1,3#], Xinni Pan[1#], Shanshan Fan[1], Cheng Cao[1], Yingao Jiao[1], Yanfei Fu[1], Jiaqi Niu[1], Shujin Lin[1], Jingmao Lao[2]*, Yanlei Liu[1,3]*

1 Institute of Intelligent Health Diagnosis and Treatment, School of Sensing Science and Engineering, School of Electronic Information and Electrical Engineering, Shanghai Jiao Tong University, 800 Dongchuan Road, Shanghai 200240, PR China

2 Gastrointestinal Surgery, The First People's Hospital of Qinzhou, Qinzhou 535000, China

3 National Engineering Center for Nanotechnology, Shanghai 200240, PR China

*Corresponding authors: Jingmao Lao, Email: 15977022288@163.com; Yanlei Liu, Email: liuyanlei@sjtu.edu.cn.

[#]contributed equally to this work.

Materials and General Methods.

All chemicals were purchased from commercial suppliers (Innochem or Acros) and used without further purification. Air-sensitive reactions were all carried out under an inert atmosphere of nitrogen or argon. Proton Nuclear Magnetic Resonance (^{1}H-NMR) spectra were recorded on Bruker BioSpin (400 MHz) spectrometer. Chemical shifts were reported as the delta scale in ppm relative to d-DMSO (δ = 2.50 ppm). High resolution mass spectrometry (HR-MS) analyses were performed using MALDI-TOF-MS techniques. X-ray Photoelectron Spectroscopy (XPS) experiments were conducted on an ESCALAB 250 (Thermo-VG Scientific). Ultraviolet-Visible-Near-Infrared (UV-Vis-NIR) absorption spectra were recorded on a DUV-3700 spectrophotometer (Shimadzu). Ultraviolet photoelectron spectroscopy (UPS) experiments were performed on Thermo Fisher Scientific, ESCALAB 250Xi. Transmission electron microscopy (TEM) images were acquired using a JEM-ARM 200F Atomic Resolution Analytical Microscope operating at an accelerating voltage of 200 kV. Dynamic light scattering (DLS) spectra were recorded on a Brookhaven Omni particle size analyzer.

Synthesis of PPAPA.

1,2,4,5-tetraaminobenzene tetrahydrochloride (TAB, 500 mg, 1.76 mmol) and 4,5,9,10-pyrenetetrone (PT, 461 mg, 1.76 mmol) were added to a 100-mL flask containing 40 mL N-methylpyrrolidone (NMP) under an argon atmosphere. The flask was placed in an ice bath (0 °C) and 0.5 mL of sulfuric acid was added slowly via a syringe. The mixture was stirred at room temperature for 2 hours before heated with stirring at 180 °C for 8 hours. After cooling down to room temperature, the reaction was quenched by

water. The resulting suspension was filtered and washed with copious amounts of water. Further purification was achieved by Soxhlet extraction for 24 h with methanol and deionized water, sequentially. The product, PPAPA, was obtained as a black powder after drying under vacuum (370 mg, 57.7% yield). IR (KBr, ν; cm^{-1}): 2920, 2852, 1660, 1404, 1300, 1113, 802, 717, and 658.

Synthesis of PPAPA NPs.

PPAPA was loaded into nanoparticles composed of DSPE-mPEG using ultrasonic nanoprecipitation method. Briefly, PPAPA (1.0 mg) was added into 1 mL of chloroform and vigorously sonicated for 30 min to disperse form solution A. DSPE-mPEG (10.0 mg) was dissolved in 1 mL of chloroform to form solution B. Solutions A and B were mixed and sonicated vigorously for 20 minutes. Then add the mixed solution to 10 mL of ultrapure water and sonicate for 10 minutes, then leave them overnight to evaporate the chloroform. The unloaded PPAPA was removed by centrifuging the supernatant solution at 15,000 rpm. Finally, add 1 mL of ultrapure water and sonicate to form PPAPA NPs, then store at 4 °C.

Photothermal Effect Evaluation

Photothermal effects were assessed by monitoring temperature changes using an IR thermal camera and analyzed by testo IRSoft. 0.2 mg/mL PPAPA (400 μL) suspension and 0.2 mg/mL SWCNT (400 μL) suspension were individually exposed to a 1064 nm laser (2.0 W/cm^2). Then, the light source was removed at approximately 220 s. Before testing, these suspensions underwent thorough ultrasonic mixing. Following previously reported method[1], the photothermal conversion efficiency was calculated according

to Eqs below:

$$\eta = \frac{hA(\triangle T_{max} - \triangle T_{max,PBS})}{I(1-10^{-A_{\lambda}})}$$

where η is the photothermal conversion efficiency, h is the heat-transfer coefficient, A is the surface area of container, T_{max} is the maximum temperature of PPAPA and SWCNT aqueous dispersion, $T_{max,PBS}$ is the maximum temperature of PBS, I is the laser power, A_{λ} is the absorbance of PPAPA and SWCNT aqueous dispersion at the wavelength of 1064 nm according to the Figure 2a.

Next PPAPA NPs aqueous solutions (400 μL) with concentrations ranging from 0.1 to 0.8 mg/mL were irradiated with 1064 nm laser (1.5 W/cm^2, 100 s). Deionized water was used as control under the same condition. Moreover, a 0.2 mg/mL PPAPA NPs solution was irradiated by the laser at a power density of 0.5, 1.0, 1.5, 2.0, and 2.4 W/cm^2 for 200 s. To investigate the photostability of PPAPA NPs, laser on/off cycle assays were carried out. A PPAPA NPs aqueous solution (0.2 mg/mL) was exposed to a 1064 nm laser (2.4 W/cm^2) for 70 s before the laser was shut off. The laser was turned on again until the solution returns to the original temperature. This cycle was repeated six times.

Cell Culture

The BEL-7402 human hepatocellular carcinoma cells and Human Liver-7702 (L02) cells were obtained from the Cell Bank of the Chinese Academy of Sciences. All cell culture supplies were provided by GIBCO (Grand Island, NY, USA). The cells were maintained in RPMI-1640 medium containing 10% fetal bovine serum (FBS) and 1%

penicillin/streptomycin. They were incubated in a humidified atmosphere at 37 °C with 5% CO_2. Cells in the logarithmic growth phase were used in all experiments.

***In Vitro* Biocompatibility *and* Photocytotoxicity**

BEL-7402 cells and L02 were cells seeded in 96-well plates at a density of 4×10^3 cells per well and cultured overnight. Then the medium was replaced by fresh medium containing varying concentrations of PPAPA NPs and the cells were incubated for an additional 24 or 48 hours. Afterward, the cells were washed three times using PBS, supplemented with fresh medium, and treated with or without NIR laser irradiation (1064 nm, 1.0 W/cm^2) for 5 minutes. After another 4 h, the standard CCK-8 assay was performed to determine the relative cell viability.

***In Vitro* Cellular Uptake**

The cellular uptake capacity of PPAPA NPs was evaluated by confocal laser scanning microscopy (CLSM, TCS SP8 STED 3X; Leica; Germany) and FACSCaliber flow cytometer (BD Biosciences, Mountain View, CA, USA). PPAPA NPs were labeled with Cy7, a red fluorescent probe, for visualization. BEL-7402 cells were seeded at a density of 2×10^5 in CLSM culture dishes and incubated at 37 °C for 24 h. The medium was then replaced with either 2 mL of fresh 1640 medium alone or 2mL of freshly prepared Cy7-PPAPA NPs solution in 1640 medium (0.2 mg/mL). The treated cells were incubated for an additional 12 h under conditions of 37 °C and 5% CO_2. Afterward, the upper supernatant was removed, and the cells were rinsed twice with 1 mL of cold PBS and stained with DAPI. The fluorescence signals of the cells were observed using CLSM. Subsequently, the cells were detached from the dishes using trypsin-EDTA. The red fluorescence signal inside cells from Cy7-PPAPA NPs was quantified via a flow cytometer.

Assessment of Photothermal Effect *In Vitro* by Live/Dead Assay and Apoptosis Assay

The photothermal effect of PPAPA NPs was scrutinized using confocal imaging and flow cytometry using the following methods. In method one, BEL-7402 cells were cultured in 48-well plates until approximately 70% confluency. The medium was then replaced with either fresh 1640 medium or a mixture medium (0.2 mg/mL) of the fresh 1640 medium and PPAPA NPs. After co-incubation for 12 h, certain wells were exposed to 1064 nm laser illumination (1.0 W/cm^2, 5 min). After 24 h of apoptosis, the cells were washed twice to remove the cell debris and stained with a viability/cytotoxicity kit according to the manufacturer's instructions. Finally, the cells were imaged using CLSM (TCS SP8 STED 3X; Leica; Germany). In method two, following 24 hours of treatment as described above, BEL-7402 cells in four sample groups were trypsinized and resuspended in 100 μL of 1 × binding buffer at a concentration of 2×10^6 cells/mL. Then, 5 μL of Annexin V−FITC and 10 μL of PI were consecutively added to the binding buffer, and the cells were incubated for an additional 10 min at room temperature in the absence of light. Finally, Cell apoptosis and necrosis were quantitatively analyzed immediately using flow cytometry.

Animal Model

All experiments using animals were carried out in accordance with the guidelines of the Animal Care and Use Committee of Shanghai Jiao Tong University. BALB/c-nude mice (age 5-6 weeks) were obtained from the Jiangsu Jicui Yaokang Biotechnology Co., LTD. The xenograft animal model was established by hypodermic injection of BEL-7402 cells suspended in 100 μL PBS into the right flank of each mouse. Mice were used for the subsequent experiments until the tumor reached a volume of approximately 100

mm^3. The tumor volume was calculated according to the following equation: $V = 0.5 \times L \times W^2$, where L and W are the longest and shortest diameters of the tumor, respectively.

In Vivo Fluorescent Imaging

The *in vivo* biodistribution of PPAPA NPs in tumor-bearing nude mice was investigated by *in vivo* fluorescence imaging. the BEL-7402-bearing mice received an intravenous injection with Cy7-PPAPA NPs and the images were captured at indicated time points (0, 2, 4, 8, and 24 h) using a PerkinElmer *in vivo* imaging system (IVIS spectrum, USA).). After completion of *in vivo* imaging, the mice were euthanized, and necropsy was performed to harvest major body organs like heart, liver, spleen, lung, kidneys, and tumor for further *ex vivo* imaging.

In Vivo Photothermal therapy (PTT)

The mice were randomly divided into four groups, each consisting of five mice: (1) Control, (2) PPAPA NPs only, (3) NIR only, and (4) PPAPA NPs + NIR. Mice belonging to groups 1 and 3 were administered sterilized PBS (100 μL/mouse) intravenously, while the remaining groups were given intravenous injections of PPAPA NPs (100 μL, 10 mg/mL). After 8 h, mice from groups 3 and 4 were anesthetized and then exposed to a 1064 nm laser for 5 min at a power density of 1.0 W/cm^2 on the tumor site. Meanwhile, an infrared camera was employed to capture *in vivo* photothermal images and record temperature fluctuations in the tumor region. After the above treatments, the tumor volume and mouse weight were observed and measured every other day. On the 15th day, mice were euthanize, and their subcutaneous tumors were excised, washed with PBS, and fixed in 4% paraformaldehyde. Finally, hematoxylin and eosin (H&E) staining and terminal-deoxynucleotidyl transferase-mediated dUTP nick-end labeling (TUNEL) staining were performed for further pathological examination.

Statistics

For both *in vitro* and *in vivo* experiments, data were gathered from at least three independent parallel trials and presented as means ± SD. Statistical significance was determined using GraphPad Prism 8.0 software (San Diego, CA, USA) through a two-tailed Student's *t*-test, with *P*-value < 0.05 regarded as statistically significant.

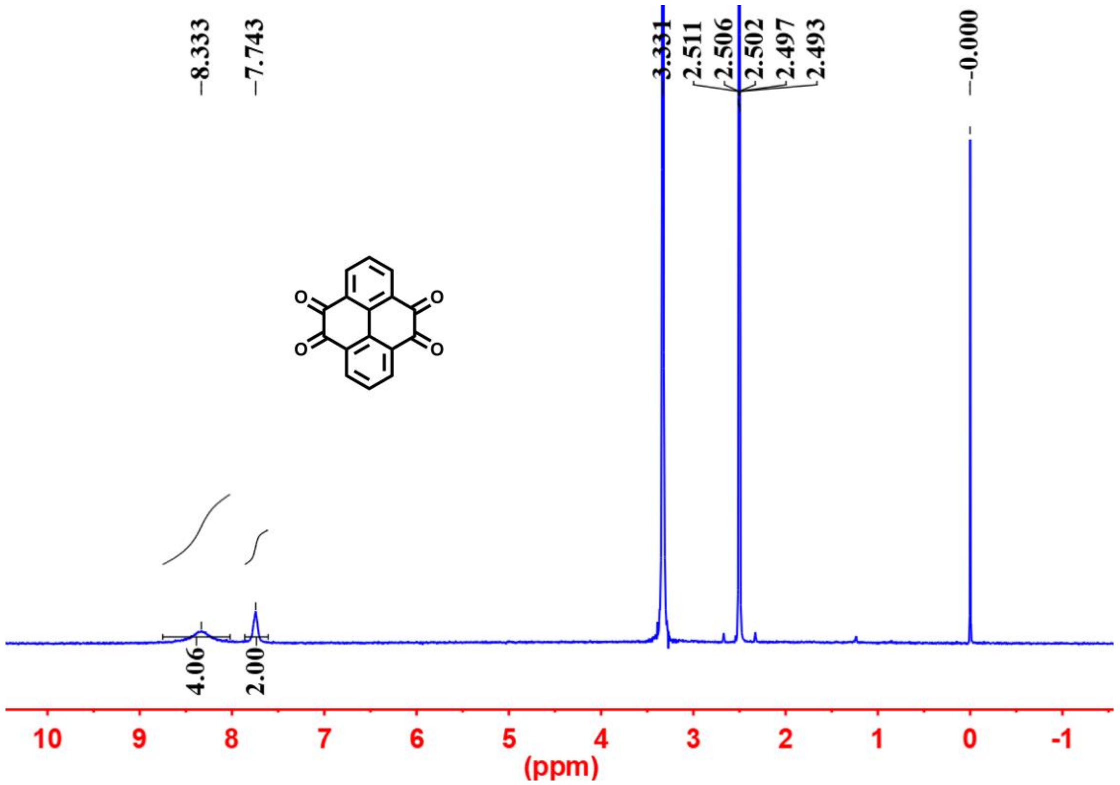

Figure S1. ^{1}H NMR spectrum of 4,5,9,10-pyrenetetrone (PT) in *d*-DMSO.

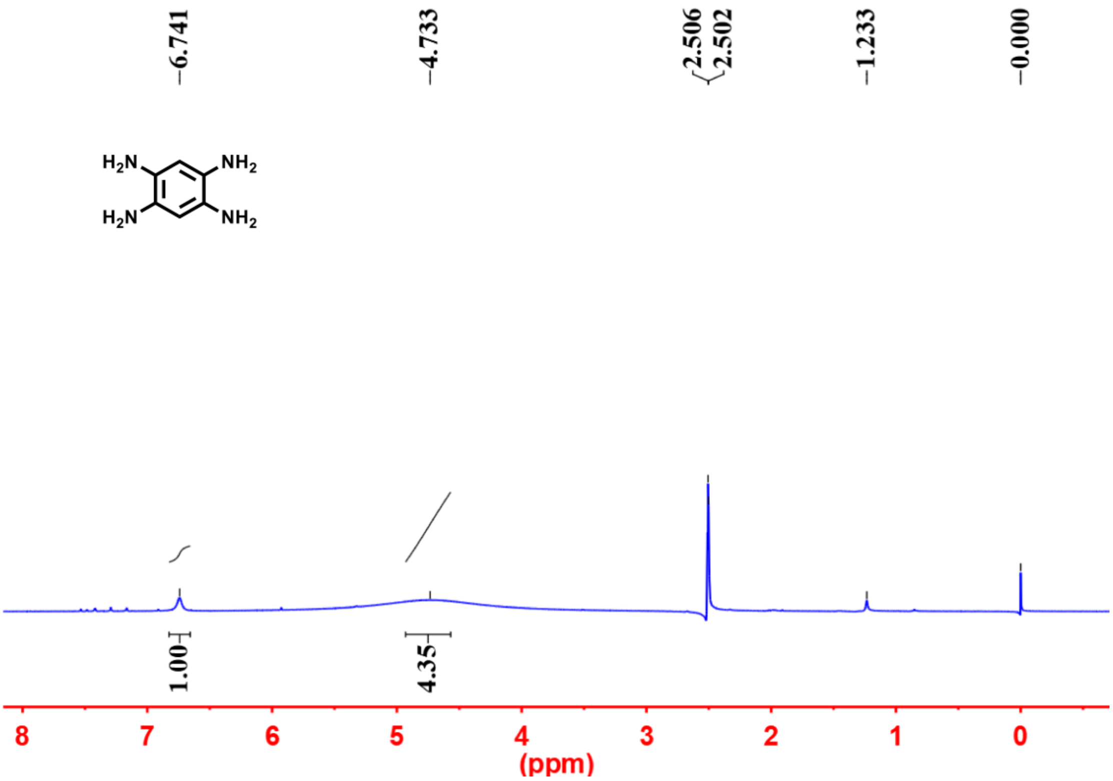

Figure S2. ^{1}H NMR spectrum of 1,2,4,5-tetraaminobenzene tetrahydrochloride (TAB) in *d*-DMSO.

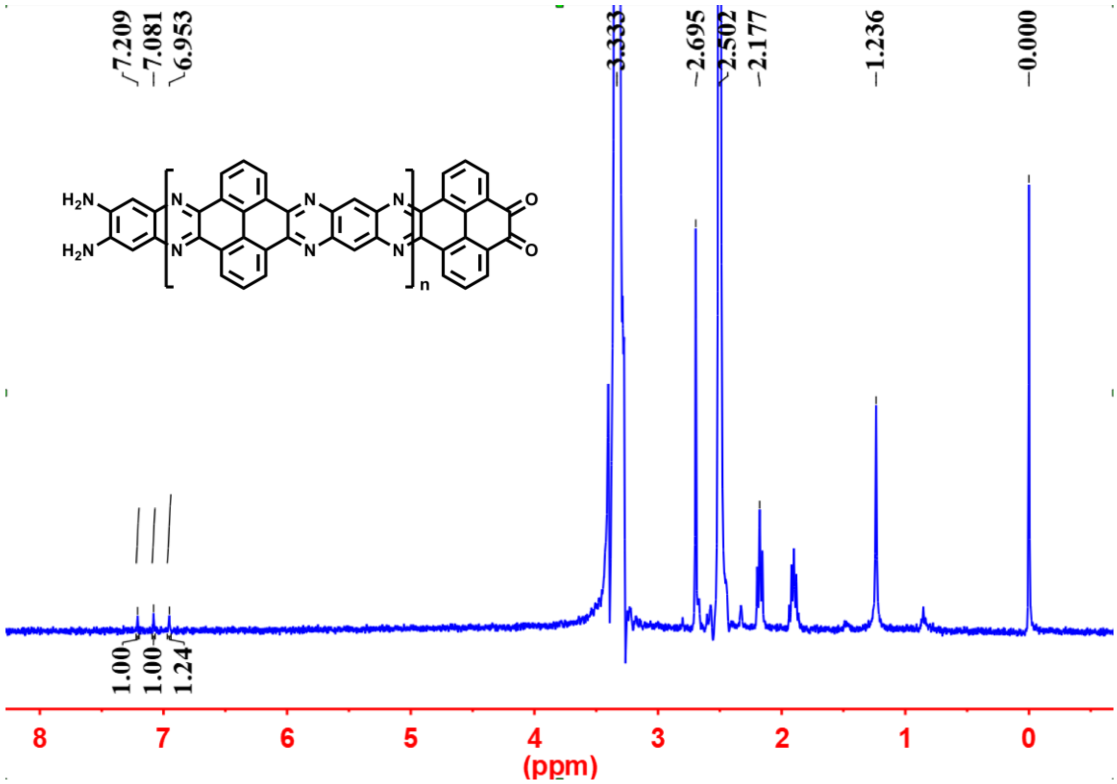

Figure S3. ^{1}H NMR spectrum of poly-phenanthrol-phenazine (PPAPA) in *d*-DMSO.

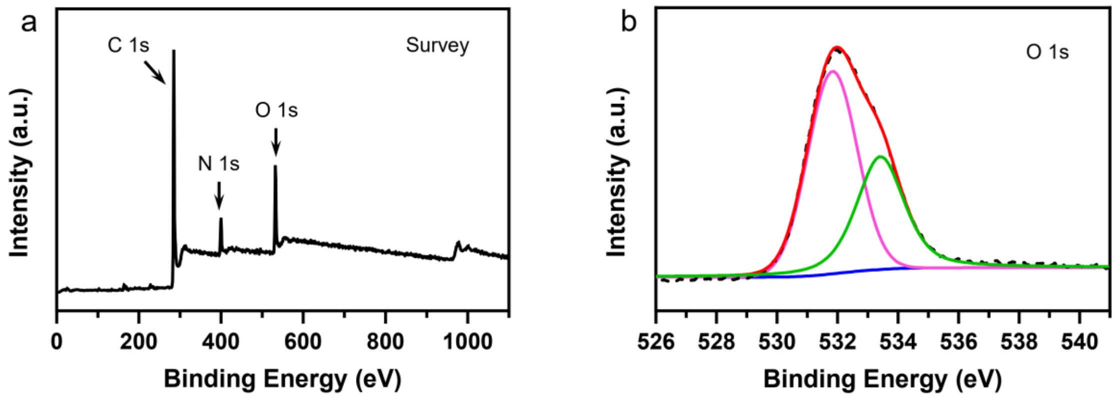

Figure S4. X-ray Photoelectron Spectroscopy (XPS) Spectra of PPAPA. (a) Full XPS spectra and (b) high-resolution O 1s XPS spectrum.

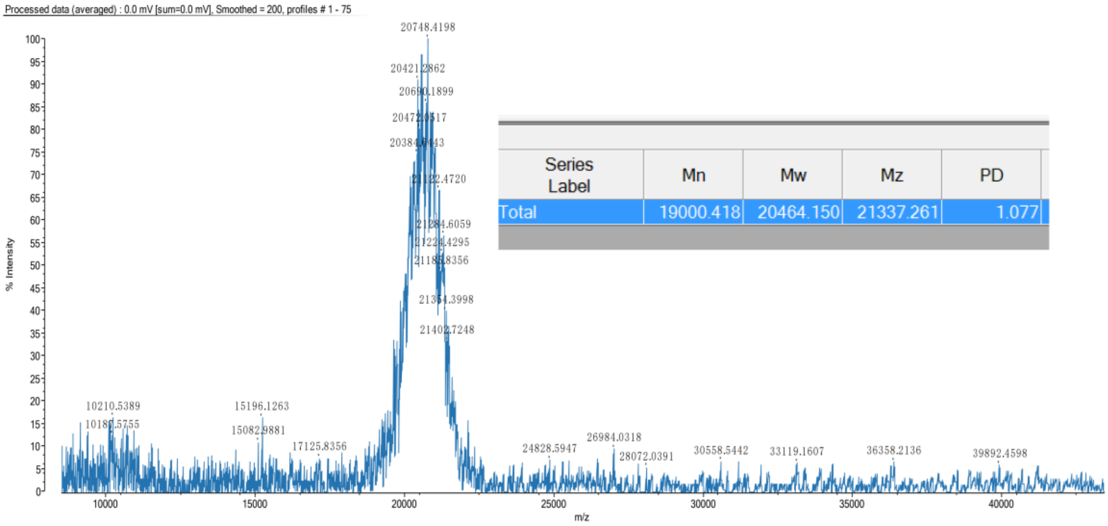

Figure S5. MALDI-TOF-MS spectrum for PPAPA.

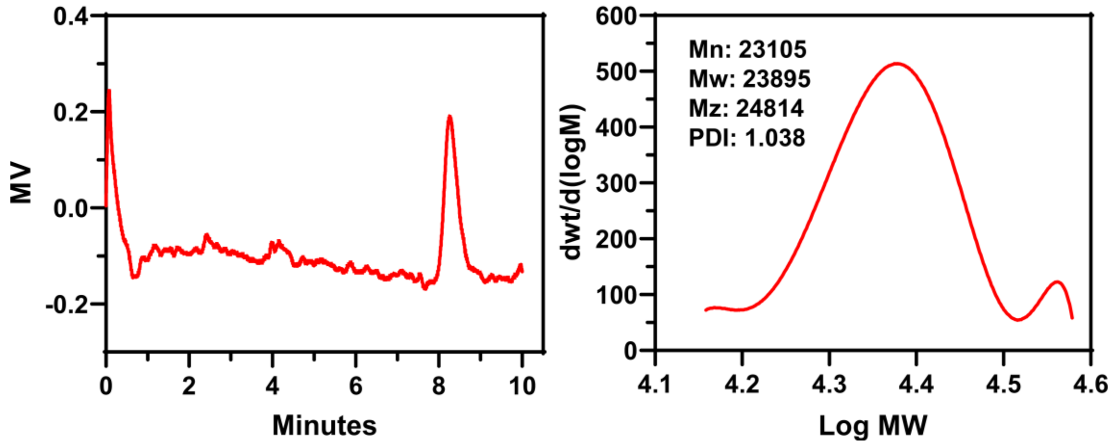

Figure S6. GPC trace and molecular weight distribution curve of PPAPA.

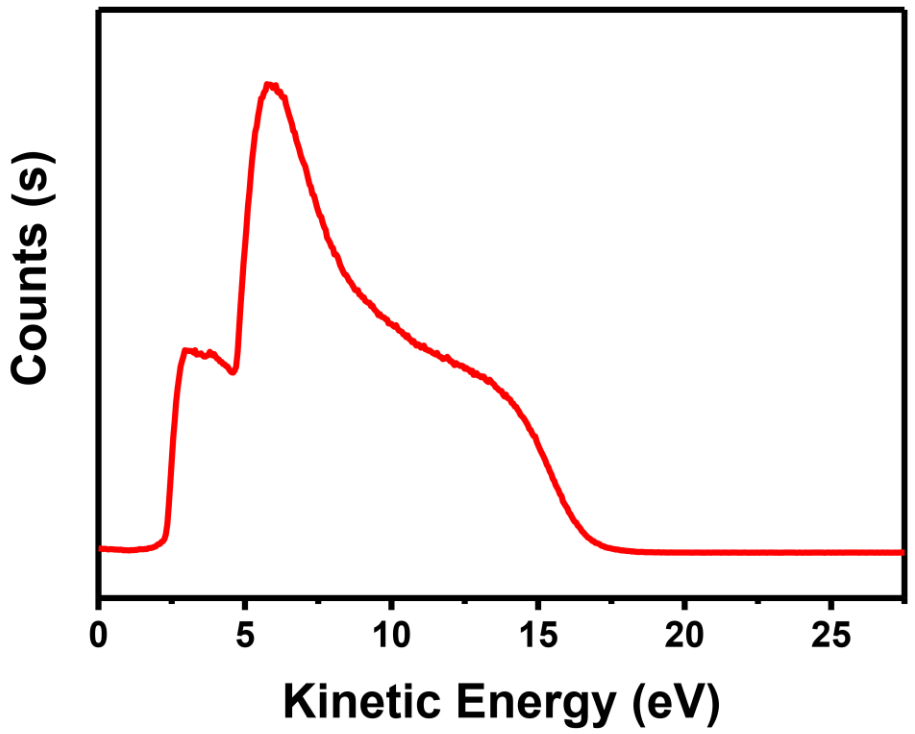

Figure S7. UPS spectra of PPAPA.

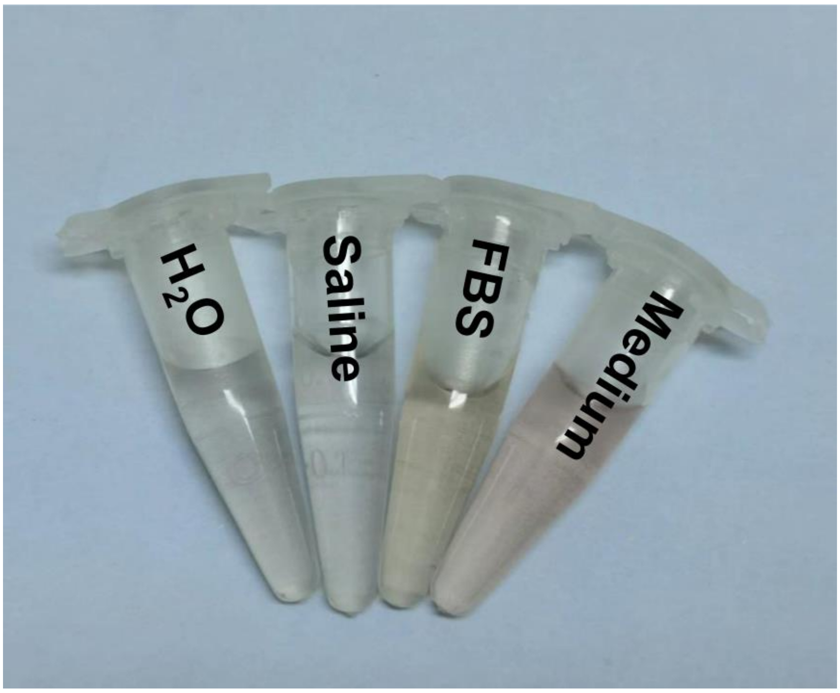

Figure S8. Photograph of PPAPA NPs dispersed in water, saline, serum and cell culture medium.

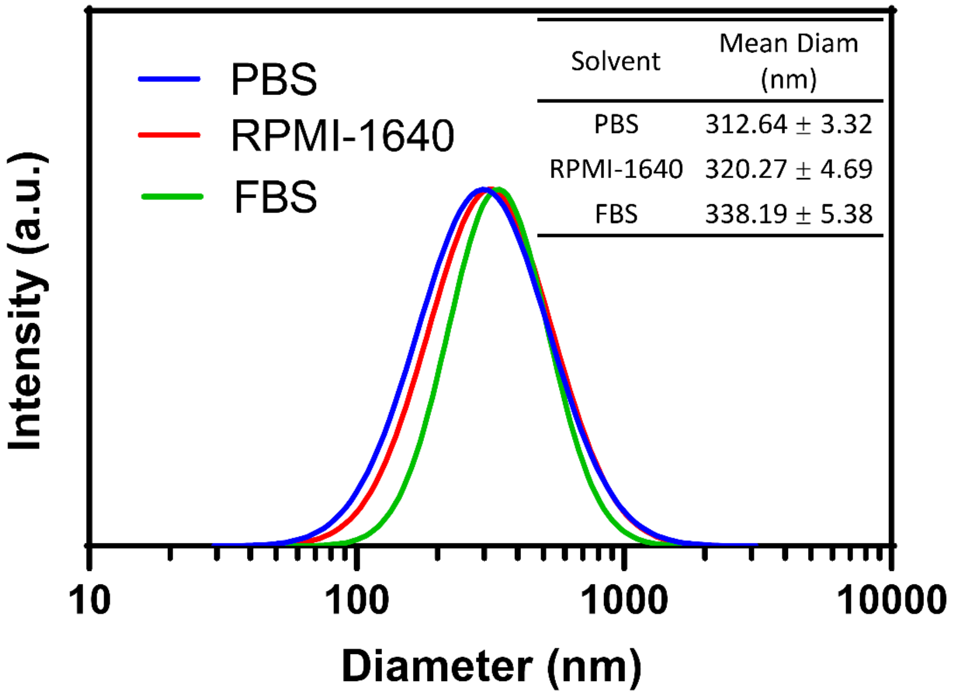

Figure S9. Dynamic light scattering (DLS) measurement of PPAPA NPs in different solvent.

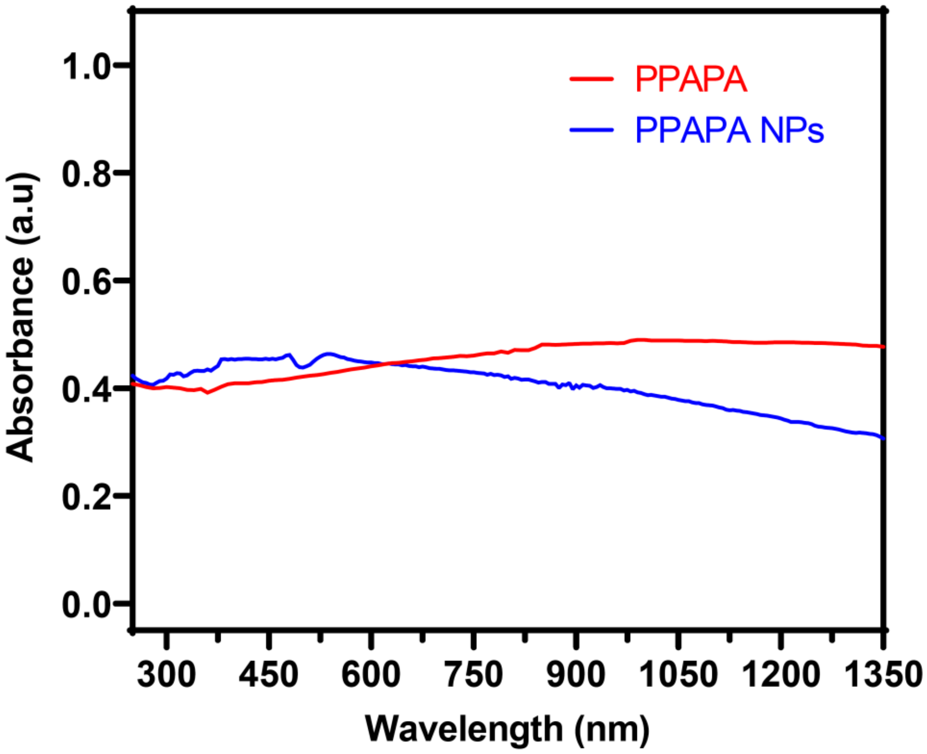

Figure S10. UV-Vis-NIR absorption spectra of PPAPA and PPAPA NPs suspensions.

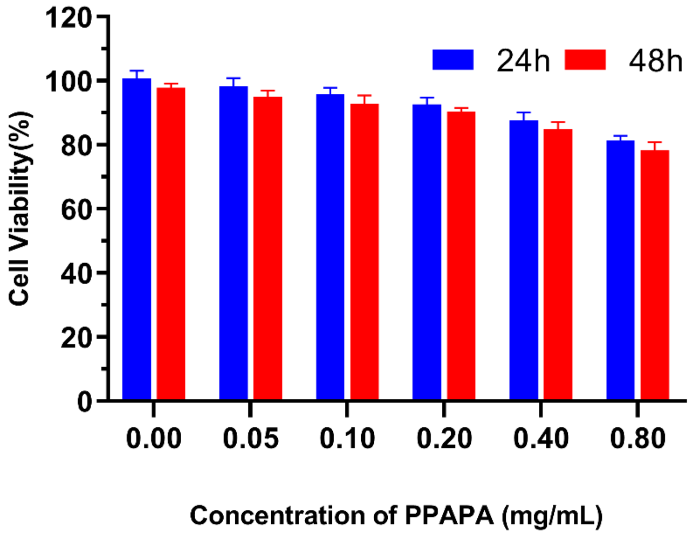

Figure S11. Cell viabilities of L02 cells incubated with PPAPA NPs at different concentrations for 24 and 48 h.

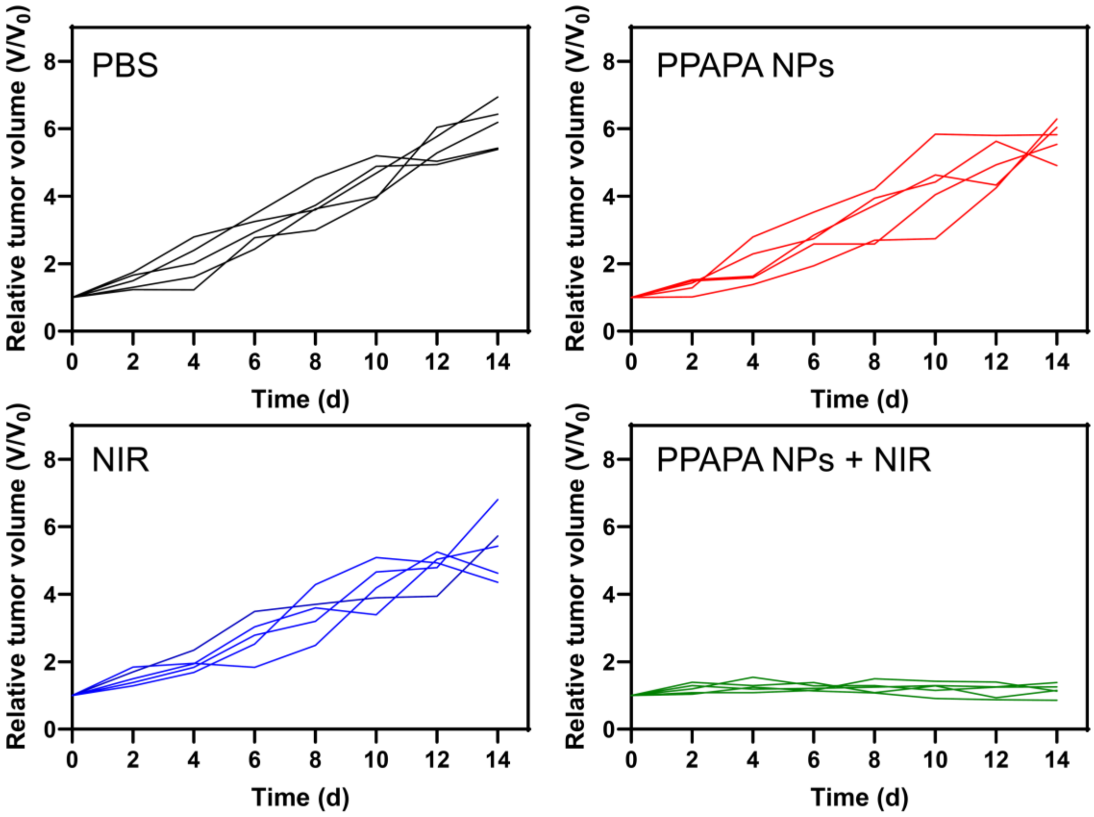

Figure S12. Tumor growth for PBS, PPAPA NPs, NIR, and PPAPA NPs + NIR groups, n= 5 per group.

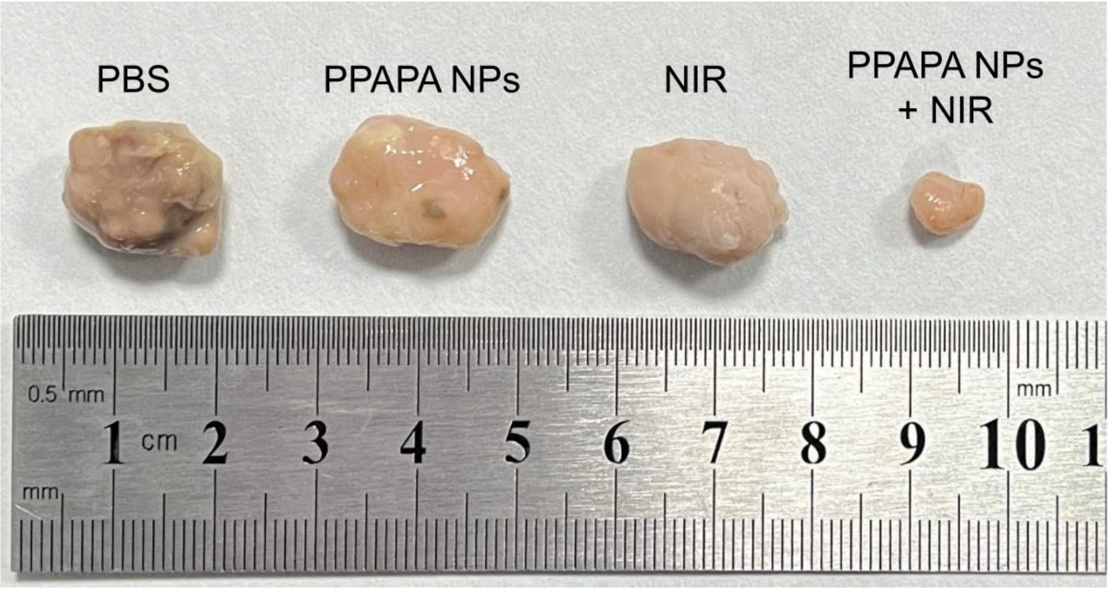

Figure S13. Representative photo of harvested tumors from mice at day 15 after different treatments.

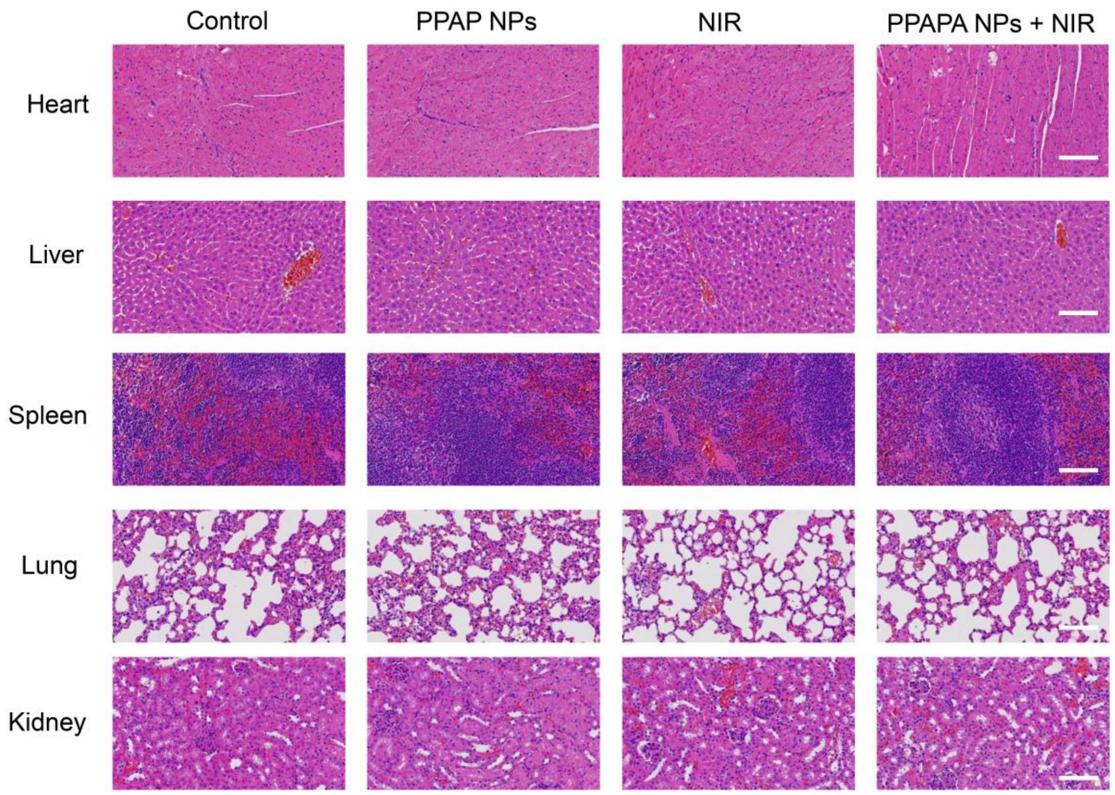

Figure S14. H&E staining of main organs after different treatment. Scale bar, 100 μm.

Reference

[1] Y.L. Liu, K.L. Ai, J.H. Liu, M. Deng, Y.Y. He, L.H. Lu, Dopamine-Melanin Colloidal Nanospheres: An Efficient Near-Infrared Photothermal Therapeutic Agent for In Vivo Cancer Therapy, Adv. Mater. 25(9) (2013) 1353-1359.http://doi.org/10.1002/adma.201204683.
